# Comprehensive analysis of global research on overactive bladder: A scientometric approach

**DOI:** 10.3389/fsurg.2022.1078052

**Published:** 2023-01-06

**Authors:** Lu Wang, Sheng Deng, Fanchao Meng, Lun Zhang, Zhenxing Min, Jitao Li, Jisheng Wang

**Affiliations:** ^1^ Department of Surgery, Beijing Xuanwu TCM Hospital, Beijing, China; ^2^ Department of Andrology, Shunyi Hospital, Beijing Hospital of Traditional Chinese Medicine, Beijing, China; ^3^ Department of Urology Surgery, The Third Affiliated Hospital of Beijing University of Chinese Medicine, Beijing, China; ^4^ Department of Andrology, Dongzhimen Hospital, Beijing University of Chinese Medicine, Beijing, China

**Keywords:** clinical research, publications, overactive bladder, analysis, bibliometric

## Abstract

**Background:**

Overactive bladder, a syndrome marked by an urgent need to urinate, is a globally prevalent ailment. Human health and quality of life are seriously affected. Therefore, it is essential to investigate the current progress and trends in this field.

**Objective:**

No bibliometric analysis of overactive bladder has been conducted. Through the use of bibliometrics and visualization, this study intends to examine the current progress and development trend of this field.

**Methods:**

Global publications on overactive bladder between January 2004 and August 2022 were extracted from the Web of Science core collection database. A bibliometric and visual analysis was carried out using VOSviewer software and CiteSpace.

**Results:**

Over the last 20 years, publications have grown rapidly, but after 2019, they started to fall. According to the collaboration network, the United States, Univ Pittsburgh AND NEUROUROLOGY AND URODYNAMICS are the most active countries, institutes AND journals in the field, respectively. All keywords were categorized by the symbiosis analysis into four categories: experimental study, symptoms, clinical use, and quality of life. The most prevalent keyword across all clusters is “overactive bladder.”

**Conclusion:**

Year after year, there have been more publications in the field of overactive bladder research in many countries, and there has been a deeper level of cooperation and exchange. Researchers will still be interested in overactive bladder in the future. Currently, the clinical application of the disease and the safety and effectiveness of medications are being investigated. However, radical innovation in relevant experimental technologies is a significant obstacle in this field.

## Introduction

OAB syndrome is characterized by the urgency of urination. Its clinical manifestations include frequent urination and nocturia with or without urinary incontinence and without urinary tract infections or other definite lesions (1–3). Relevant studies showed that OAB was a highly prevalent disease worldwide with a 17% incidence rate (4). There was no significant difference in incidence among different countries (5–8). The same incidence is seen in men and women (9). Related studies have shown that the incidence of OAB in children is about 16.6%, which also seriously affects the quality of life and brings huge losses to the families of the children (10, 11). Although numerous studies have explored OAB, its pathogenesis is still unclear and has poor clinical efficacy, thereby seriously affecting the quality of life of the patients. The first-line pharmacological therapy can worsen bowel dysfunction. A large number of studies make it difficult to predict future OAB hotspots. Therefore, it is necessary to summarize and analyze the research trends of OAB. The existing literature can help in understanding OAB and provide ideas for follow-up studies.

Bibliometrics is a statistical and quantitative analytical tool for literature-based research. It can find the correlations between disciplines and subdisciplines by analyzing keywords, authors, countries, and institutions and grasp the current hot issues in academic studies (12). Moreover, it can also predict the developmental direction of disciplines (13). Web of Science database is currently the most commonly used database for bibliometric analysis (14). VOSviewer and CiteSpace are the most commonly used software for bibliometric analysis (15–17). Using bibliometric methods, this study aimed to identify the contributions, research contents, and emerging trends in countries, institutions, and authors in the field of OAB studies.

## Methods

### Data sources and retrieval strategies

The existing technology can not realize the analysis of multi-language and multi-database literature. The Web of Science database contains a relatively comprehensive collection of OAB-related literature. Web of Science database was searched to collect the OAB-related studies from its inception until August 1, 2022. The database source was limited to the Web of Science Core Collection (WoSCC), and only research and review articles published in the English language were selected. The main search terms were as follows: overactive bladder, bladder hyperactivity, urinary bladder overactive, and bladder overactivity disorder. All the data from the Web of Science, which met the criteria, were downloaded for further bibliometric analysis.

### Statistical analyses

Before analyzing the data, it was cleaned up to remove the meaningless keywords and merged those having the same meaning. VOSviewer (1.6.16) was used to identify the production countries/regions, research institutions, journals, and authors, and the main co-cited journals, authors and references, and related visualization networks were constructed. The cluster analysis of the high-frequency keywords was performed using the cluster function in CiteSpace (5.0.R2). The data management and analysis of publications were performed using Microsoft Office Excel 2019. In the VOSviewer network map, the size of nodes reflected the number of studies or co-occurrence frequency, the links between the nodes represented the co-occurrence relationship, and the size of the links represented the co-occurrence frequency of the two nodes. The Impact factors (IFs) of academic journals were collected from the 2021 Journal Citation Reports (JCR) (Clarivate Analytics, Philadelphia, PA, USA).

## Results

### Annual growth trend of global publications over the years

**Figure 1:**
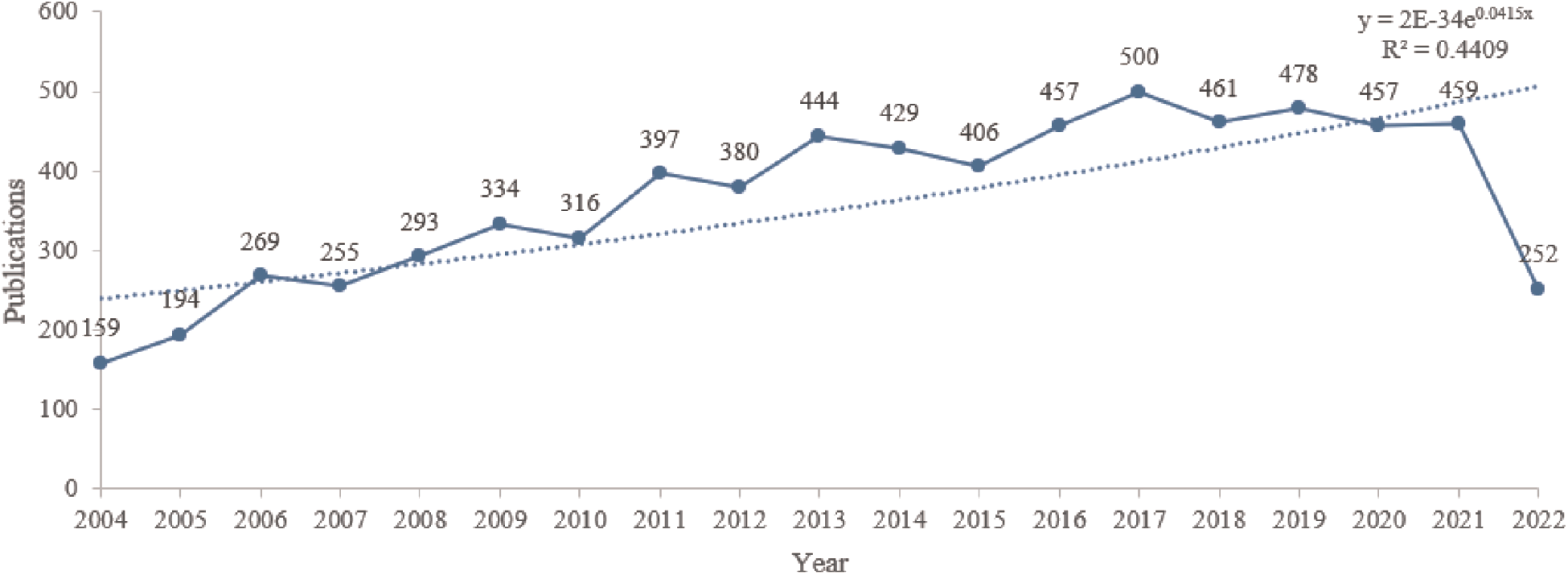
Trend of publications in original human studies on overactive bladder.

A total of 11,241 papers published between January 1, 2004, and February 1, 2022, were included for analysis in this study. The data were cleared using CiteSpace software, excluding 4,301 literature records, such as solicits and statements, which were unrelated to the subject. Finally, a total of 6,940 articles were included. The annual growth trend of the cumulative number of publications is shown in Figure 1.

### Countries-based analysis of publications

**Figure 2:**
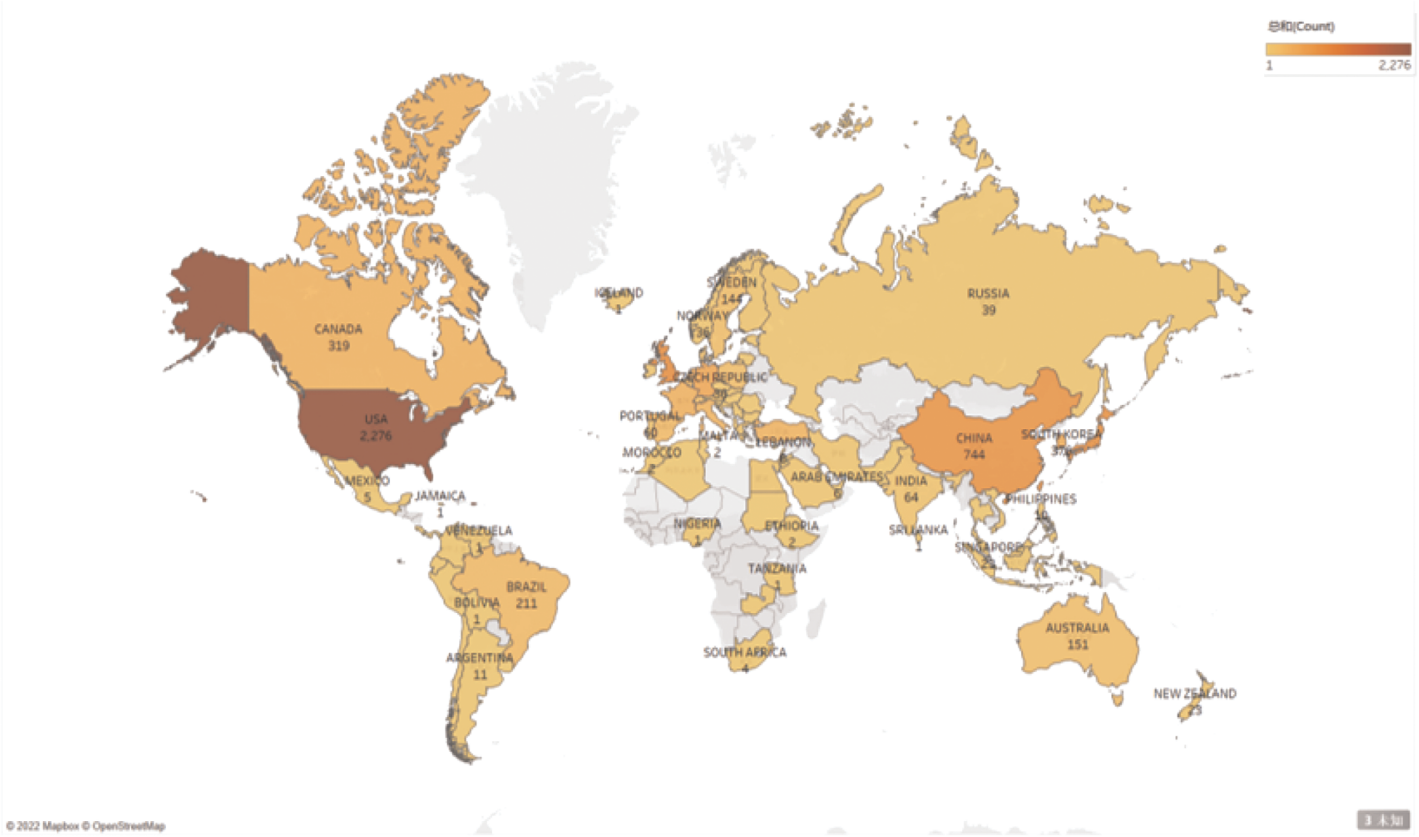
Geographic map illustrating the origin of publications in scientific research on overactive bladder in original human studies.

From January 2001 to August 2022, the United States (US) ranked first in the OAB-related articles with 2,276 studies, followed by the United Kingdom (UK) (824 articles) and Japan (766 articles). The number of citations in the US (68,965 citations) was also significantly higher compared to that in the other countries, followed by the UK (31,751 citations) and the Netherlands (13,808 citations). Currently, the number of research articles on OAB in the US are ahead of the other countries. Figure 2 shows the regional concentration of the study area. The top 10 countries based on the number of published articles and citations are listed in Table 1.

**Table 1:** Top 10 countrys publishing original human studies on the topic of overactive bladder.

Rank	Country	Publications	% of 6940	Rank	Country	Total citations
1	USA	2276	32.80%	1	USA	68,965
2	Britain	824	11.87%	2	Britain	31,751
3	Japan	766	11.04%	3	Netherlands	13,808
4	China	744	10.72%	4	Japan	13,009
5	Germany	467	6.73%	5	Germany	12,840
6	Netherlands	408	5.88%	6	China	10,703
7	South korea	376	5.42%	7	Italy	10,122
8	France	327	4.71%	8	Canada	9181
9	Canada	319	4.60%	9	Sweden	8152
10	Turkey	300	4.32%	10	France	7772

**Figure 3:**
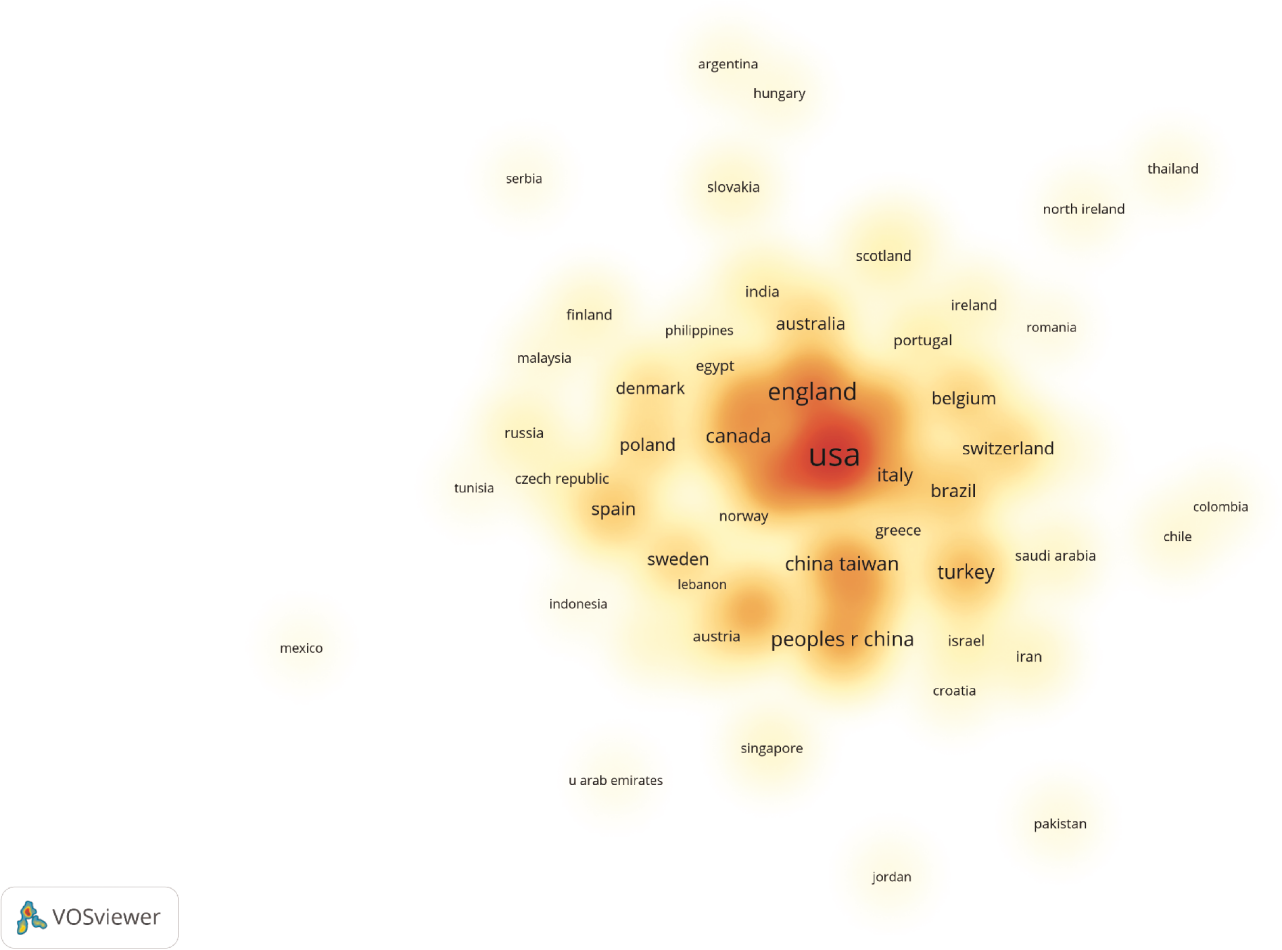
Network of institutions visualized in VOSviewer; the size of circles reveals the number of publications in different countries.

**Figure 4:**
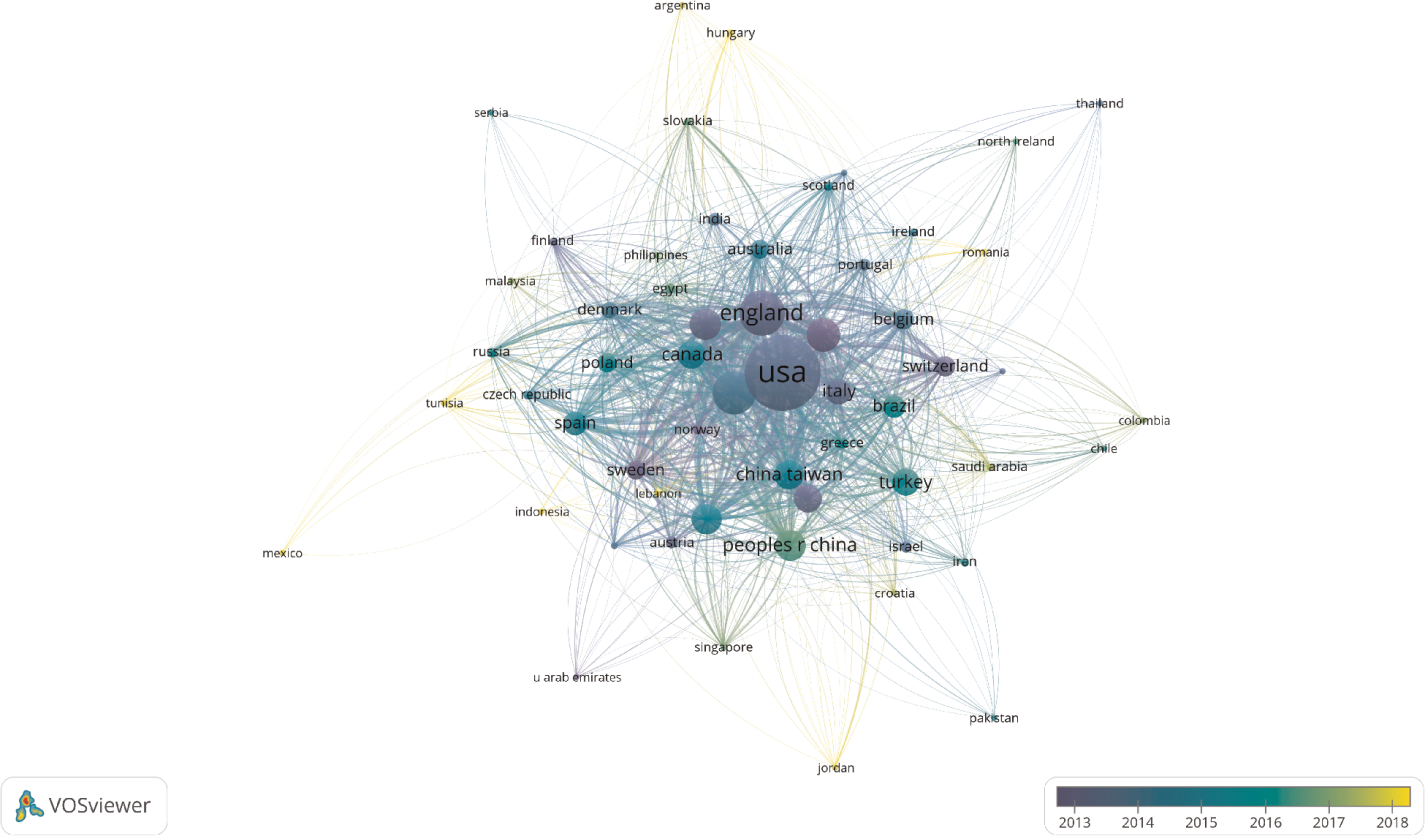
Network map illustrating international research collaborations between countries in original human studies on overactive bladder. Colors represent the date of publication.

**Figure 5:**
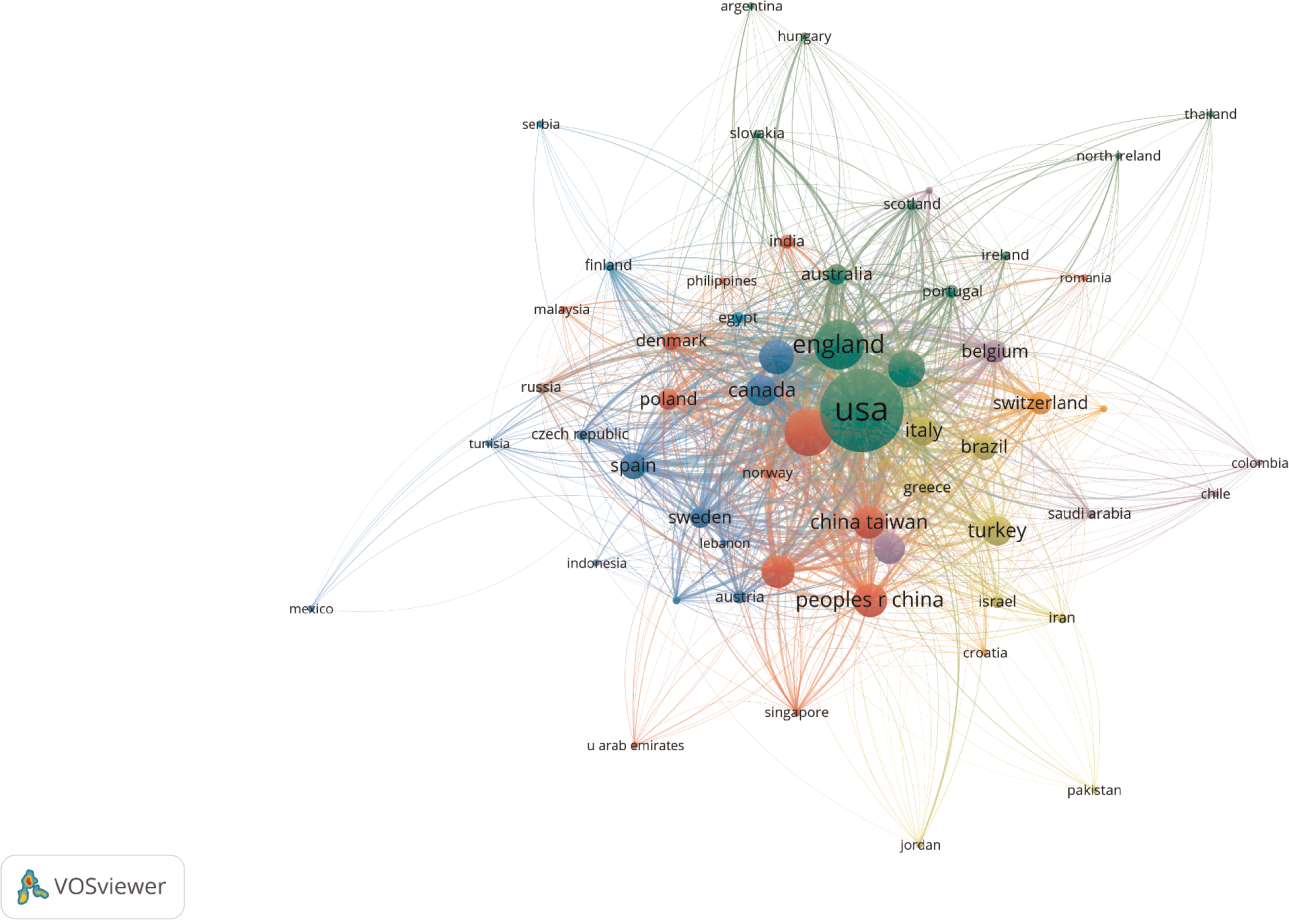
Network map illustrating international research collaborations between countries in original human studies on overactive bladder; the size of circles reveals the number of publications.

The number of publications from a particular country is shown in Figure 3. The size of the circle indicates the number of publications; the larger the circle, the more the number of publications. The number of recent publications was high, especially in the US (Figure 4). The light-colored circles represent new publications, showing that more articles were published on OAB by the US in recent years. The cooperative relationship between countries could also be observed. The US has the largest number of publications, and other countries had a strong cooperative relationship with the US (Figure 5).

### Institutions-based analysis of publications

University of Pittsburgh ranked first by publishing 221 articles on OAB, followed by Pfizer Inc. with 184 publications and University of Toronto with 106 publications (Table 2). It could be seen from the Table 2 that the US dominated the research in this field, with a huge number of articles and citations.

**Table 2:** Top 10 institutions publishing original human studies on the topic of overactive bladder.

Rank	Institutions	Publications	Original country	Rank	Institutions	Total citations	Original country
1	Univ Pittsburgh	221	USA	1	Univ Pittsburgh	11,987	USA
2	Pfizer Inc	184	USA	2	Pfizer Inc	7,484	USA
3	Univ Toronto	106	Canada	3	Univ N Carolina	5,466	USA
4	Sungkyunkwan Univ	98	Korea	4	Royal Hallamshire Hosp	5,330	Britain
5	Tzu Chi Univ	97	China-tw	5	Univ Toronto	4,821	Canada
6	Vanderbilt Univ	97	USA	6	Southmead Hosp	4,607	Britain
7	Duke Univ	94	USA	7	United BioSource Corp	4,023	USA
8	Univ Penn	88	USA	8	Johannes Gutenberg Univ Mainz	3,505	Germany
9	Chang Gung Univ	87	China-tw	9	Vanderbilt Univ	3,069	USA
10	Buddhist Tzu Chi Gen Hosp	86	China-tw	10	Gothenburg Univ	2,945	Sweden

### Journals-based analysis of publications

As shown in Table 3, more than 20% of the total OAB-related articles were published in the top three journals (21.30%). In terms of the number of publications, Neurourology and Urodynamics (IF = 2.367) ranked first with 701 articles, followed by Journal of Urology (IF = 7.6). Most of the journals were closely related to urology, while other types of journals appeared less frequently.

**Table 3:** Top 10 journals publishing original human studies on the topic of overactive bladder.

Rank	Journals	Publications	Citescore (2021)	SJR quartile
1	Neurourology and Urodynamics	701	2.367	q3
2	Journal of Urology	427	7.6	q1
3	International Urogynecology Journal	350	1.932	q4
4	Bju International	294	5.969	q1
5	Urology	280	2.633	q3
6	Luts Lower Urinary Tract Symptoms	163	1.374	q4
7	European Urology	139	24.267	q1
8	International Journal of Urology	131	2.896	q3
9	Female Pelvic Medicine “and” Reconstructive Surgery	105	1.913	q4
10	International Neurourology Journal	104	3.038	q2

### Top 10 articles based on the number of citations

The top 10 articles with the total number of citations are listed in Table 4. “Population-based Survey of urinary incontinence, Overactive bladder, and other lower urinary tract symptoms in five countries: “Results of the EPIC Study” was the most published and cited, with 1,699 citations. Followed by STOPP (Screening Tool of Older Person's Prescriptions) and START (Screening Tool to Alert Doctors to Right) from Ireland Treatment). Consensus validation “, citation 913 times.Five of the top 10 articles belonged to the US. Therefore, the US was a major source of high-quality articles. Two of the top 10 articles belonged to the Greece. The other top 10 articles belonged to Ireland, England, and Japan.

**Table 4:** Top 10 cited papers on the topic of overactive bladder.

Rank	Title	Year	Corresponding authors	Journal	Total citations
1	Population-based survey of urinary incontinence, overactive bladder, and other lower urinary tract symptoms in five countries: Results of the EPIC study	2006	Irwin, Debra E	European Urology	1699
2	STOPP (Screening Tool of Older Person's Prescriptions) and START (Screening Tool to Alert Doctors to Right Treatment). Consensus validation	2008	O'Mahony, D	International Journal of Clinical Pharmacology and Therapeutics	913
3	EAU Guidelines on the Treatment and Follow-up of Non-neurogenic Male Lower Urinary Tract Symptoms Including Benign Prostatic Obstruction	2013	Gravas, Stavros	European Urology	785
4	Update on AUA Guideline on the Management of Benign Prostatic Hyperplasia	2011	McVary, Kevin T	Journal of Urology	750
5	Activation of Human Brown Adipose Tissue by a beta 3-Adrenergic Receptor Agonist	2015	Cypess, Aaron M	Cell Metabolism	612
6	Worldwide prevalence estimates of lower urinary tract symptoms, overactive bladder, urinary incontinence and bladder outlet obstruction	2011	Irwin, Debra E	Bju International	608
7	The effects of antimuscarinic treatments in overactive bladder: An update of a systematic review and meta-analysis	2008	Chapple, Christopher R	Bju International	561
8	The impact of overactive bladder, incontinence and other lower urinary tract symptoms on quality of life, work productivity, sexuality and emotional well-being in men and women: results from the EPIC study	2008	Coyne, Karin S	European Urology	559
9	EAU Guidelines on the Assessment of Non-neurogenic Male Lower Urinary Tract Symptoms including Benign Prostatic Obstruction	2015	Gravas, Stavros	European Urology	544
10	Symptom assessment tool for overactive bladder syndrome- Overactive bladder symptom score	2006	Homma, Yukio	Urology	456

### Keywords analysis

**Figure 6:**
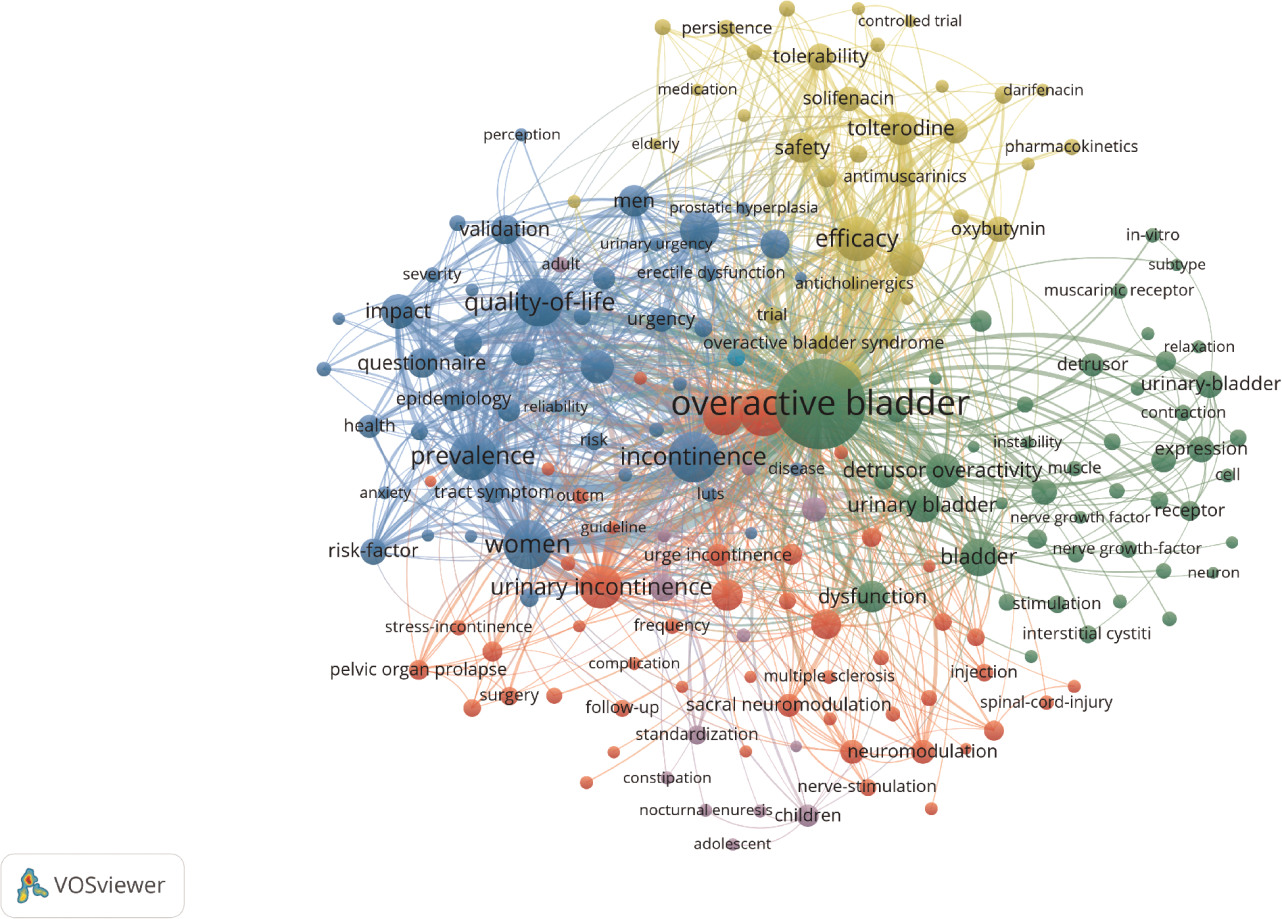
Mapping of the keywords in the field of overactive bladder. Different colors represent different clusters.

As shown in Figure 6, the different clusters are represented by different colored circles as follows: experimental studies (red circles), symptoms (blue circles), clinical medication (yellow circles), and quality of life (green circles).

**Figure 7:**
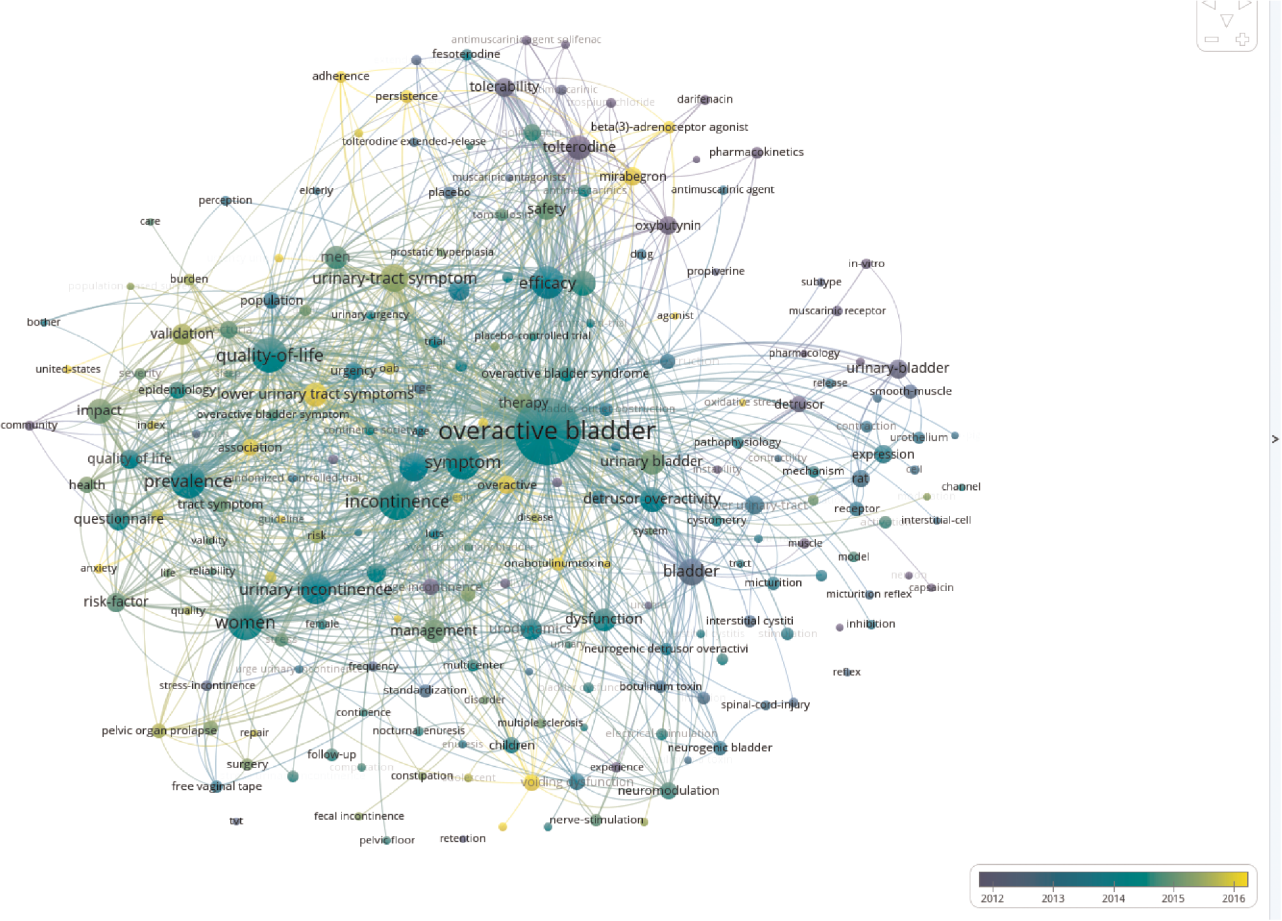
Co-occurrence analysis of all keywords in the publications onOveractive bladder: mapping of the keywords in the field of overactive bladder. Distribution of keywords according to the average time of appearance; blue represents an early appearance, and yellow represents a late appearance.

As shown in Figure 7, the keywords tagged by VOSviewer are colored to indicate different years of appearance (AAY). Specifically, the blue color indicated the relatively early appearance of the keyword, and the yellow color indicated a more recent appearance.

**Figure 8:**
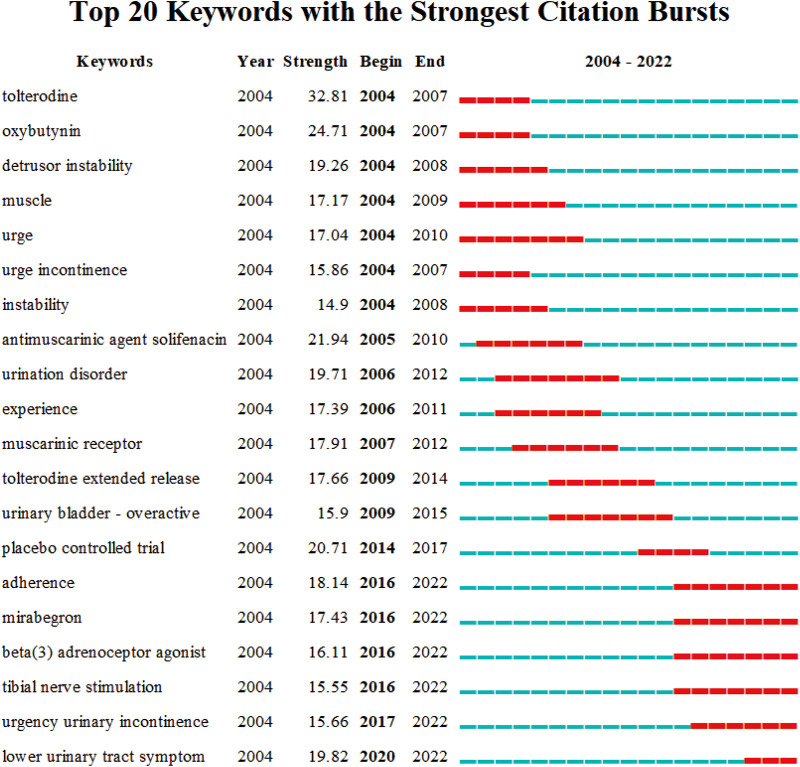
Top 20 keywords cited the most frequently from 2004 to 2022 and have received continuous attention for a period of time. The red bars represent frequently cited keywords during this time period, and the green bars represent infrequently cited keywords.

Among all the keywords, 20 keywords had the longest duration (Figure 8). The latest highlighted keywords included “lower urinary tract symptom” and “association”, which were highlighted for approximately 3 years from 2020 to 2022. “Trospium chloride” lasted the longest (9 years) from 2004 to 2013, indicating that this term had been the center of focus in previous studies. Since 2020, the keywords “lower urinary tract symptom” and “association” have gradually become the center of research focus conducted in OAB.

## Discussion

### Research trends in OAB studies

Despite numerous bibliometric studies related to urinary system, those conducted on OAB are lacking. To the best of our knowledge, this was the first study, performing bibliometric and visual analysis for OAB. VOSviewer, CiteSpace, and other visualization methods were used for the analysis of important published information, such as country, year, institution, and keywords.

The number of relevant publications had generally been increasing year by year since 2004, but decreased in 2019 (Figure 1). A population survey conducted on urinary incontinence, OAB, and other lower urinary tract symptoms in five countries (18) accelerated the number of publications since 2006 and paved the way for OAB research. In addition, two studies conducted on the adverse effects of OAB on the quality of life and clinical management were published in 2008, which accelerated the studies on OAB since then (19, 20). All these studies appeared among the top 10 articles (Table 4), indicating that the OAB studies received widespread attention. However, the research interest declined in 2019. We initially thought that the phenomenon was affected by Covid-19. After reviewing a large number of relevant literatures, we found that most of them did not have significant changes before and after 2019. There is a lack of strong evidence on the impact of Covid-19 on related scientific research. Combined with the hot keywords after 2019, we believe that the lack of recent breakthrough research advances in the OAB field is a major factor limiting the number of publications. The participation of experts from other fields as well as well-developed research strategies and novel techniques are needed to overcome the current research barriers. To overcome the current research obstacles, it is necessary to refer to the research process of other diseases with relatively clear pathogenesis, analyze the relevant data of OAB combined with the field of information technology, and find new research directions from a macro perspective.

The US was more advanced in OAB research, which was reflected in its highest number of articles, citation (Table 1). Among the top 10 institutions, contributing the most articles in this field, five institutions were affiliated in the US, among which, University of Pittsburgh ranked first with more in-depth studies on OAB and far more contributions than the other four institutions (Table 2). The OAB-related studies at University of Pittsburgh mainly focused on the clinical diagnosis and treatment of OAB patients, novel therapeutic drugs, and technologies as well as explored the pathogenesis of OAB (21, 22). The US gives great importance to scientific research and invests a lot of money and manpower in the medical field. Moreover, the US has advanced medical equipment and scientific research environment and is a world leader in research in many fields, such as global research trends in prostate disease, erectile dysfunction, and premature ejaculation (23–25). In addition, the studies on OAB in the US started early and published the most frequently cited review article (Table 4). The current study summarized the previous studies on OAB, visually analyzed the hotspots and shortcomings in this field, and laid a foundation for further exploring the complex mechanism of OAB.

The cooperative relationships of countries (Figure 5) indicated that the US and the UK had a greater international influence. The US worked with many countries. In contrast, the scope and close international cooperation of the UK were inferior to those of the US. The visual analysis of relevant data indicated that the studies on OAB were concentrated in developed countries. The differences in economic development level and national policies might lead to different degrees of research attention in this field. The developed countries with high Gross Domestic Product have more advanced research technologies and better scientific research platforms, which make their scientific studies more reliable and rank their research output higher. The scientific productivity of a country determines the level of international cooperative relationships of that country. In combination with the previous bibliometric studies, international collaborations might be one of the strongest predictors of scientific productivity. Therefore, countries and institutions should strengthen international exchanges and cooperation.

The journal contribution and citation analyses can reflect the attention of researchers to a research field. Currently, the OAB-related studies are relatively concentrated, and the top 10 journals mainly included those based on urology research. Among them, only one journal had an IF >10, and European Urology had an IF of 24.267. This indicated that the researchers' attention to this field required further strengthening.

### Studies focused on OAB

The high-frequency keywords in bibliometrics and visual analysis could be roughly divided into several OAB-related categories (Figure 6): experimental studies (red circles), symptoms (blue circles), clinical medication (yellow circles), and quality of life (green circles). “Overactive bladder” was located at the center of visualization and was closely related to other categories of keywords, thereby playing a crucial role in this study. Among the top 10 articles (Table 4), three were related to epidemiological investigations of OAB. The studies by Irwin, DE et al., conducted in Canada, Germany, Italy, Sweden, and the UK, showed an 11.8% overall prevalence of OAB. The prevalence was similar in men and women and increased with age. It was expected that the number of people affected by the disease would increase in the future, especially in developing countries (18, 26). There are four OAB-related clinical guidelines and expert consensus for the clinical treatment of OAB (27–30).

As shown in Figure 8, the keywords were sorted in chronological order and the changes in keywords were mainly concentrated from 2012 to 2016. There was no significant change in keywords from 2012 to 2016. Based on the number of articles published over the years, the studies on OAB were more active from 2012 to 2016. There was no significant improvement in both the number of publications and research content. Moreover, after 2016, the novel research contents were also fewer. At present times, the research contents are still stuck on the aspects of epidemiology, symptoms, clinical medications, etc. The detailed pathogenesis and experimental research contents are rare and require further studies by relevant researchers. The current research directions are also relatively limited. Therefore, researchers must find high-quality research directions as well as new methodologies in the future.

Analyzing the occurrence frequency of the top 20 keywords by burst detection (Figure 8) showed that “trospium chloride” had the longest research heat in nearly the past two decades. Trospium chloride is a quaternary ammonium compound with anticholinergic and antispasmodic effects (31). The parasympathetic blockade of the drug can relax the bladder's smooth muscle and increase its capacity. The top 20 keywords mainly emerged from 2004 to 2010. The hot keywords mainly included “tolterodine”, “oxybutynin”, and other commonly used clinical drugs and “detrusor instability”, “hyperreflexia”, and other pathogeneses. From 2010 to 2014, the research contents mainly focused on the impact of “tolterodine” on OAB. After 2016, the research direction shifted towards the effects of “mirabegron” and “lower urinary tract symptom” in this field, indicating the changes in people's cognition of OAB. In combination with “oxybutynin”, “trospium chloride”, and “mirabegron”, “Tolterodine” also appeared in the top 20 keywords, indicating that the focus of OAB-related studies in the past two decades on clinical drug treatment. Mechanistic studies focused on the function of the detrusor. This suggested that OAB was still a difficult clinical disease, and researchers were still studying more effective clinical drugs.

Among the top 10 articles (Table 4), two were epidemiological investigations of OAB. Studies showed that OAB was a common disease worldwide having the greatest impact in Asia. The number of people affected by the disease might gradually increase in the future, especially in developing countries. Three clinical guidelines for OAB provide guidance for its clinical diagnosis, differential diagnosis, treatment, and nursing. Based on the publication time of the article, it could be seen that novel drug therapies and technologies continuously appear, which might impact the treatment plan and lead to constant advancements in the clinical studies of OAB.

The keywords in Figure 8 represent the progress in the OAB-related studies in the past two decades, showing an overall cooperative network. After paying attention to the disease, the experts begin to study the clinical drugs for the treatment of OAB as well as the safety and effectiveness of drugs. This also improved the epidemiological investigation of the disease. The pathogenesis of the disease has been preliminarily explored. However, Figure 8 indicates that the current relevant studies lack new directions. Therefore, new research directions and therapeutic drugs are urgently needed. In addition, overactive bladder is more common in children, seriously affecting the quality of life of children. The symptoms of overactive bladder in children and adults are not completely consistent, but there is a close association (32). Although anti-muscarinic agents are considered the mainstay of OAB drug therapy, only two drugs, Oxybutynin and Propiverine, are currently approved for use in the pediatric population (33). The literature on children with overactive bladder is limited and needs further research.

However, there are still some shortcomings in this study. The current study searched for the OAB-related articles in the Web of Science database and obtained relatively objective search results. However, this study did not highlight the advantages of this strategy. Moreover, this study focused on the collection and analysis of OAB-related articles published in the English language, thereby eliminating those published in other languages, which might lead to limiting the current results. In addition, this study only retrieved the articles published in the past two decades, and those published before 2004 were not included, which might also impair the current analyses. Future studies should focus on more data bases, different languages, and different periods. Other databases, such as Medline, Scopus, or Google Scholar, could be adopted for this purpose. Moreover, exploring studies published in the last 30 years or older would enrich the current results.

## Conclusions

This study provided a comprehensive assessment of the global OAB-related research trends and priorities using bibliometric and visual analysis and provided ideas for future research directions. In the past 20 years, the number of OAB-related articles in various countries increased year by year, and international cooperation and exchange between countries have been deepened. However, the OAB-related studies have decreased in recent years. The US has made the highest contribution in this area. OAB might be of interest to researchers in the future. However, in the past 20 years, the hot keywords of publications focus on clinical aspects, and there is no essential change, and the number of published literatures grows at a gentle rate. OAB research is stuck in a rut. Therefore, breakthrough innovation in relevant experimental technologies should be a priority challenge.

## Data Availability

The raw data supporting the conclusions of this article will be made available by the authors, without undue reservation.
